# EGFR endocytosis is a novel therapeutic target in lung cancer with wild-type EGFR

**DOI:** 10.18632/oncotarget.1711

**Published:** 2014-01-16

**Authors:** Ukhyun Jo, Kyong Hwa Park, Young Mi Whang, Jae Sook Sung, Nam Hee Won, Jong Kuk Park, Yeul Hong Kim

**Affiliations:** ^1^ BK21 Plus program, Korea University Anam Hospital, Seongbuk-gu, Seoul, Republic of Korea; ^2^ Department of Oncology/Hematology, Korea University Anam Hospital, Seongbuk-gu, Seoul, Republic of Korea; ^3^ Department of Pathology, Korea University Anam Hospital, Seongbuk-gu, Seoul, Republic of Korea; ^4^ Deapartment of Radiation Cancer Research, Korea Institute of Radiological and Medical Sciences, Nowon-Gu, Seoul, Republic of Korea

**Keywords:** EGFR, endocytosis, gefitinib, lung cancer, Rab25

## Abstract

Oncogenic alterations of epidermal growth factor receptor (EGFR) signaling are frequently observed in lung cancer patients with worse differentiation and poor prognosis. However, the therapeutic efficacy of EGFR-tyrosine kinase inhibitors (TKIs) is currently limited in selected patients with EGFR mutations. Therefore, in this study, we investigated the potential molecular mechanism that contributes to cell viability and the response of gefitinib, one of the EGFR-TKIs, in lung cancer models with wide-type EGFR (wtEGFR). Interestingly, we found that EGF-induced EGFR endocytosis is existed differently between gefitinib-sensitive and -insensitive lung cancer cell lines. Suppressing EGFR endocytos decreased cell viability and increased apoptotic cell death in gefitinib-insensitive lung cancer with wtEGFR in vitro and in vivo. In addition, we found that Rab25 was differentially expressed in between gefitinib-sensitive and -insensitive lung cancer cells. Rab25 knockdown caused the changed EGFR endocytosis and reverted the gefitinib response in gefitinib-sensitive lung cancer with wtEGFR in vitro and in vivo. Taken together, our findings suggest a novel insight that EGFR endocytosis is a rational therapeutic target in lung cancer with wtEGFR, in which the combined efficacy with gefitinib is expected. Furthermore, we demonstrated that Rab25 plays an important role in EGFR endocytosis and gefitinib therapy.

## INTRODUCTION

Epidermal growth factor receptor (EGFR) is a transmembrane growth factor receptor with tyrosine kinase (TK) activity that controls cell proliferation and survival [1, 2]. Cellular activity of EGFR is dysregulated by several mechanisms including gene mutation, copy number variation, and protein overexpression [3, 4]. Oncogenic alterations in EGFR are frequently reported in patients with lung cancer and are implicated in the pathogenesis of the disease such as increased tumor proliferation, poor differentiation, and a worse prognosis, indicating that EGFR is an important therapeutic target in lung cancer [4-7]. Based on previous reports, EGFR-targeted drugs have been developed as two major types: monoclonal antibodies (mAbs) [8] and small-molecule tyrosine kinase inhibitors (TKIs) [9, 10]. mAbs act by binding to the EGFR extracellular region and compete with its ligands, whereas TKIs were designed to reversibly bind the ATP-binding site of the cytoplasmic kinase domain of EGFR, thereby inhibiting its TK activity.

Gefitinib is a selective EGFR-TKI and was the first approved for clinical use as an orally administered drug for patients with lung cancer [9, 11]. The malfunction in EGFR TK activity caused by gefitinib results in obstruction of cellular signaling mediated mainly through the RAS-RAF-MEK-ERK and the PI3K-PTEN-AKT pathways. This eventually leads to decreased cell proliferation and increased apoptosis [12]. Apoptotic cell death induced by gefitinib is accompanied by G0/G1 cell cycle arrest with down-regulation of CDKs/cyclins [13] and anti-apoptotic proteins [12]. Moreover, gefitinib alone or in combination with other chemotherapy shows clinically meaningful antitumor activity in patients with advanced lung cancer [14-16]. However, the therapeutic efficacy of gefitinib increases noticeably in patients with lung cancer who have somatic mutations in the EGFR TK domain such as deletions in exon 19 or a point mutation (L858R) in exon 21 [17, 18]. However, the frequency of patients with EGFR mutations in unselected lung cancer is 10-40%, depending on the ethnicity of the study population [19, 20]. In other words, most of patients with lung cancer have wild-type EGFR (wtEGFR) which generally exhibits no response to gefitinib treatment [21]. Nevertheless, 10 ~ 20% patients with lung cancer without EGFR mutations therapeutically benefit from gefitinib, implying that EGFR mutations may not be the sole determinants of gefitinib efficacy [22, 23]. However, the potential molecular mechanism that expands the clinical benefit of gefitinib in patients with lung cancer and wtEGFR remains unclear.

Binding of ligands to EGFR induces translocation of EGFR from the plasma membrane to the intracellular region independently of its phosphorylation-mediated TK activation [24, 25]. Ligand-induced EGFR endocytosis is mainly mediated by clathrin- and non-clathrin mediated endocytosis [26]. In particular, dynamin, which is a guanosine 5'-triphosphatase (GTPase), functions as a key regulator in both EGFR endocytic pathways [27]. Furthermore, the change in EGFR endocytosis caused by dynamin depletion attenuates EGFR activation and degradation. [28]. Actually, EGFR endocytosis and its endosomal-mediated sorting are thought to be a cellular mechanism to induce degradation and termination of activated EGFR signaling or as a recycling mechainsm to return to the cell surface for continued signaling [29]. However, some reports show that the intracellular translocation of EGFR regulates the EGFR signaling pathway, consequently affecting cell growth and survival [30, 31]. The impaired EGFR endocytic pathway is implicated in carcinogenesis, as it potentially results in uncontrolled signal transduction [24, 32]. However, the therapeutic relevance of EGFR endocytosis in lung cancer has not been investigated.

In this study, we examined the phenotypic and molecular differences between gefitinib -sensitive and -insensitive lung cancer cells with wtEGFR using *in vitro* and *in vivo* models and found a potential relationship between gefitinib response and EGFR endocytosis. We also demonstrated that suppressing EGFR endocytosis could aeffect cell viability and the gefitinib response in gefitinib-insensitive lung cancer with wtEGFR. Additonally, we also confirmed that Rab25 is associated with EGFR endocytosis and the gefitinib response.

## RESULTS

### Effects of gefitinib on cell survival and EGFR signaling in lung cancer cells with wtEGFR

**Figure 1:**
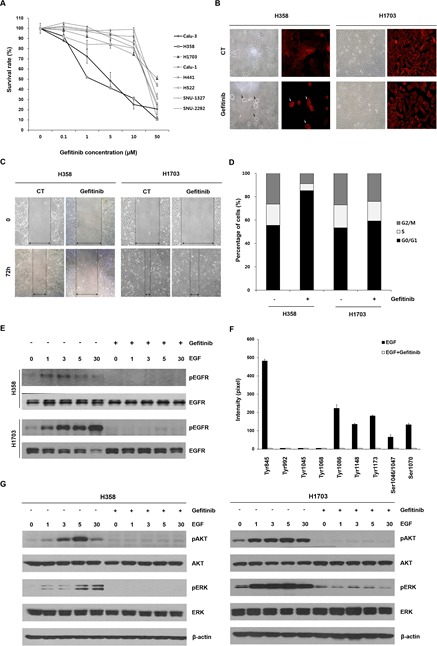
Effects of gefitinib in lung cancer cells with wtEGFR (A) Changes in cell survival caused by gefitinib were determined using MTT assay. The cells were treated with the indicated concentrations of gefitinib for 72 h. Each data point represents mean results of six independent determinations with the standard error (SE). (B) Morphological changes caused by gefitinib were representatively analyzed in H358 and H1703 cells using light microscopy and red fluorescent-conjugated phalloidin staining. After a 48 h incubation with or without gefitinib (10 μM), cell images were captured at a magnification of 200×. Black and white arrows indicate the morphologically changed cells (C) Changed cell motility capacity was evaluated using a wound healing assay. The cell images after treatment with gefitinib (10 μM) were captured at a magnification of 100×. (D) Redistributed percentages of the cell cycle caused by gefitinib were evaluated after PI staining and flow cytometry. Data represent mean results acquired from three independent experiments. (E) Effects of gefitinib on epidermal growth factor (EGF)-induced epidermal growth factor receptor (EGFR) phosphorylation were examined using Western blot analysis. The cells were pretreated with gefitinib (10 μM) under serum-free conditions for 24 h and incubated for further indicated times in the presence or absence of 100 ng/ml EGF. (F) Statuses of multiple phosphorylation sites of EGFR in H1703 cells were determined using an antibody array. Relative expression levels of phosphorylated EGFR sites were calculated with a gel doc image analyzer. The ratio of phosphorylated EGFR was normalized to total EGFR acquired from three experiments in the same membrane. Each bar is the SE. (G) The effects of gefitinib on EGF-induced phosphorylation of ERK and AKT were evaluated by Western blot analysis.

We first proflied gefitinib response in the eight lung cancer cell lines (H1703, Calu-1, H441, H522, SNU-1327, SNU-2292, H358 and Calu-3) with wtEGFR. Six of the eight lung cancer cell lines (H1703, Calu-1, H441, H522, SNU-1327 and SNU-2292) were relatively insensitive to gefitinib (IC_50_>10 μM) compared to the other two lung cancer cell lines (H358 and Calu-3) (IC_50_<10 μM) (Figure 1A). To examine further differential effects of gefitinib between these lung cancer cell lines, H358 and H1703 cells were chosen as gefitinib-sensitive and -insensitive cells, respectively. In the following comparative experiments, H358 cells exhibited morphological changes, retarded wound healing and G0/G1 arrest of the cell cycle after gefitinib treatment but H1703 cells did not show any changes in cell phenotype following gefitinib treatment (Figure 1B-D). We next investigated whether the differential effects of gefitinib between these lung cancer cell lines were associated with activation status of the EGFR signaling pathway. Interestingly, phosphorylation of EGF-induced EGFR was inhibited by gefitinib treatment in both H358 and H1703 cells regardless of the gefitinib response (Figure 1E). In gefitinib-insensitive H1703 cells, we also profiled the activation statues of multiple EGFR phosphorylation sites that regulate various downstream cellular signaling pathways. As shown in Figure 1F, seven of ten phosphorylation sites (Tyr845, Tyr1086, Tyr1148, Tyr1173, Ser1046/1047, and Ser1070) were activated by EGF stimulation, but all EGFR phosphorylated sites were blocked by gefitinib treatment. Moreover, phosphorylation of AKT and ERK, well-known downstream molecules in the EGFR signaling pathway, was also inhibited by gefitinib in both H358 and H1703 cells (Figure 1G). Similar results were detected in the other six lung cancer cell lines (Data not shown). These results suggest the existence of an unknown mechanism that regulates the response to gefitinib in lung cancer with wtEGFR.

### EGFR endocytosis is associated with the gefitinib response in lung cancer with wtEGFR

**Figure 2:**
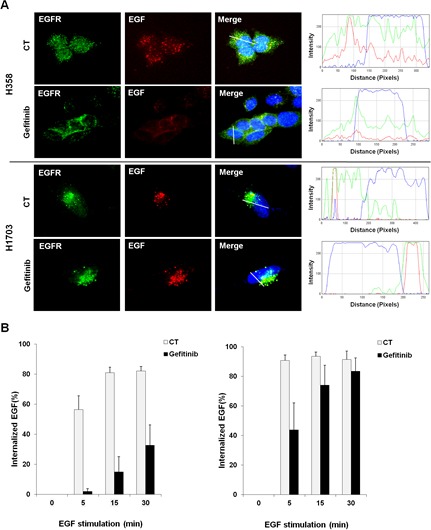
Corelation between EGFR endocytosis and gefitinib response in lung cancer cells with wtEGFR (A) The difference in EGFR cellular distribution after gefitinib treatment was profiled by immunoflurescence staining. Cells were pretreated with gefitinib (10 μM) under serum-free conditions for 24 h and incubated for further 30 min with Texas Red conjugated EGF (100 ng/ml). After staining with EGFR antibody and DAPI, immunofluorescence images were obtained using a fluorescence microscope at a magnification of 400×. Plot of intensity values along a white line segment in each immunofluorescence image was analyzed using Image J; red (EGF), green (EGFR) and blue (DAPI). (B) Changes in EGFR endocytosis were monitored using fluorescent-conjugated EGF. After incubation with Alexa Fluor 488-conjugated EGF (500 ng/ml) and gefitinib (10 μM), the percentage of internalized EGF was examined by flow cytometry. Each bar represents mean values acquired from three experiments with standard error.

Some reports show the possibility that ligand-induced internalization of EGFR, one of its degradation processes, is a potential molecular mechanism that controls cellular signaling independent of its kinase activity [25, 29, 33]. Therefore, we investigated whether the cellular distribution of EGFR correlates with the different responses to gefitinib between gefitinib-sensitive and -insensitive cells using immunofluorescence staining. As shown in Figure 2A, EGFR was localized within the intracellular region forming a punctate structure after EGF stimulation in both H358 and H1703 cells. However, this spatial change of EGFR induced by EGF stimulation was suppressed after gefitinib treatment in gefitinib-sensitive H358 cells but not in gefitinib-insensitive H1703 cells. The distinguished profile of EGFR distribution between these two lung cancer cell lines was confirmed by fluorescence intensity plot analysis (Figure 2A, right panel) and in other lung cancer cell lines (Supplemental Figure S1).

To further examine whether the differential status of EGFR distribution between gefitinib-sensitive and -insensitive cells resulted from dysregulation of EGFR endocytosis, we determined the frequencies of EGF-induced EGFR endocytosis by flow cytometry with fluorescent-conjugated EGF. As shown in Figure 2B, the frequency of internalized EGF in H358 cells increased gradually in a time-dependent manner and then decreased after gefitinib treatment. In contrast, EGF internalization in H1703 cells was not reduced after gefitinib treatment compared to that of H358 cells (Figure 2B). These results suggest that EGFR endocytosis may be one of potential mechanisms to explain the different responses to gefitinib between gefitinib-sensitive and -insensitive cells in lung cancer with wtEGFR.

### Antitumor effects by inhibiting EGFR endocytosis *in vitro* and *in vivo*

**Figure 3:**
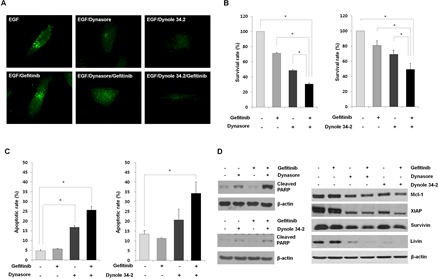
Evaluation of the anti-proliferative effects of EGFR endocytosis inhibitors (A) Inhibition of of EGFR endocytosis was induced by treatment with dynasore and dynole 34-2, or a combined treatment with gefitinib in H1703 cells. The altered internalization of EGFR endocytosis was measured by immunofluorescence staining with a specific antibody against EGFR. The immunofluorescence images were obtained using a fluorescence microscope at a magnification of 400×. (B) Effects of EGFR endocytosis inhibitors alone or combination with gefitinib on cell viability were analyzed by the MTT assay. The cells were treated with dynasore (100 μM) or dynole 34-2 (10 μM) or in combination with gefitinib (10 μM) for 48 h. Each bar represents mean values acquired from three experiments with standard error (SE). * p < 0.05. (C) The rate of apoptosis was investigated by Annexin V-FITC staining. The percentages of Annexin V-positive cells were examined by flow cytometry under the same conditions as the MTT assay. Each bar represents mean values acquired from three experiments with SE. * p < 0.05. (D) Changed expression levels in apoptosis related proteins were evaluated after treatment with EGFR endocytosis inhibitors and gefitinib using Western blot analysis. Cell lysates were blotted for the indicated proteins with the appropriate antibodies after cell harvest.

Based on previous results, we hypothesized that regulating EGFR endocytosis could reverse the response to gefitinib in gefitinib-insensitive cells. To test the hypothesis, we used two inhibitors of EGFR endocytosis; dynasore and dynole 34-2, which are known potent inhibitors of dynamin, a key molecule in the EGFR endocytic pathways. As shown in Figure 3A, EGF-induced EGFR spatial changes were effectively blocked after treatment with dynasore or dynole 34-2 in gefitinib-insensitive H1703 cells. Moreover, dynasore or dynole 34-2 significantly reduced cell viability in combination with gefitinib compared to those in treatment with the drugs or gefitinib alone (Figure 3A and Supplemental Figure S2). Next, we examined the frequencies of apoptotic cells and expression statues of apoptosis-related proteins to evaluate whether the decreased cell viability was related to apoptotic cell death. As shown in Figure 3C, treatment with either dynasore or dynole 34-2 caused apoptosis in gefitinib-insensitive H1703 cells and the proportion of apoptotic cells increased substantially in a combined treatment with the EGFR endocytosis inhibitors and gefitinib. Moreover, the increased apoptotic cell death induced by the EGFR endocytosis inhibitors was accompanied by upregulation of cleaved PARP and down-regulation of Mcl-1, survivin, XIAP and livin (Figure 3D). The expression changes in these apoptosis-related proteins were higher in the combined treatment with EGFR endocytosis inhibitors and gefitinib than that of the other single treatment.

**Figure 4:**
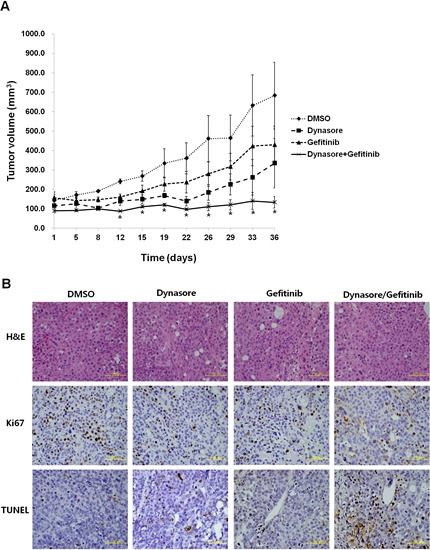
Antitumor effects in combination with EGFR endocytosis inhibitor and gefitinib in a xenograft mouse model (A) Gefitinib-insensitive SNU-1327 cells were inoculated subcutaneously and the mice were treated with DMSO, dynasore (30 mg/kg, 2 days/week), gefitinib (5 mg/kg, 4 days/week), dynasore (30 mg/kg) plus gefitinib (5 mg/kg) intraperitoneally for 3 weeks, and mean tumor volume with standard error were plotted. * p < 0.05. (B) Hematoxylin and eosin (H&E), Ki67, and TUNEL staining of representative tissue sections from tumors in each mouse group. Yellow scale bar is 100 μm.

We then evaluated the anti-tumor efficacy of an EGFR endocytosis inhibitor *in vitro* using a mouse xenograft model. Gefitinib-insensitive SNU-1327 cells were injected subcutaneously into athymic nude nu/nu mice. When the average size of tumors reached 100-200 mm^3^, the mice were treated intraperitoneally with either dynasore or gefitinib alone, or a combined treatment of dynasore and gefitinib. As shown in Figure 4A, mice treated with the combination of dynasore and gefitinib showed significantly the most inhibited efficacy of tumor formation compared to mice treated with each drug alone, and showed the same results as those of observed *in vitro*. In addition, we observed that the lowest number of proliferating cells and highest number of apoptotic cells were found in tumors from mice treated with the combination of dynasore and gefitinib (Figure 4B). These findings suggest that suppressing EGFR endocytosis can play a role to overcome the therapeutic limitation of gefitinib in lung cancer with wtEGFR.

### Rab25 expression is related to EGFR endocytosis and the gefitinib response in lung cancer with wtEGFR

**Figure 5:**
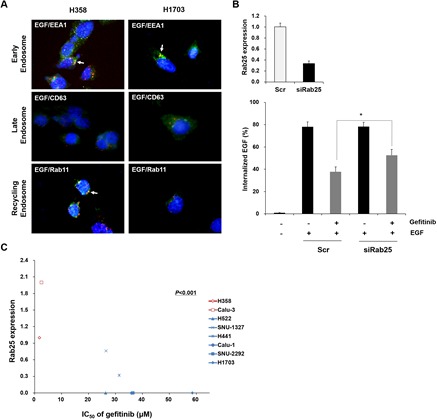
Status of Rab25 expression is associated with EGFR endocytosis and gefitinib response (A) Difference in endosomal localization of EGF-induced EGFR between H358 and H1703 cells was profiled by immunofuorescence staining with the indicated antibodies. The immunofluorescence images were obtained using a fluorescence microscope at a magnification of 400×. Each white arrow indicates co-localization (yellow). (B) Effect of Rab25 expression on EGFR endocytosis was examined in siRab25 transfected H358 cells by flow cytometry. After siRab25 or scrambled (Scr) transfection, the cells were incubated with gefitinib (10 μM) and the percentage of internalized Alexa Fluor 488-conjugated EGF (500 ng/ml) was analyzed. Each bar represents mean values acquired from three experiments with standard error (SE). * p < 0.05. (C) Correlation between Rab25 expression and gefitinib response was determined by comparative analysis. Expression of Rab25 mRNA was evaluated in eight lung cancer cell lines with wtEGFR using quantitative RT-PCR. Relative Rab25 expression values were normalized to values of GAPDH. IC_50_ of each cell was obtained from MTT assay results. The P value represents statistical comparisons between Rab25 expression and gefitinib response. Red: gefitinib-sensitive cell lines. Blue: gefitinib-insensitive cell lines.

EGFR endocytosis is a complex cellular process which includes many modulating proteins [29]. Therefore, EGFR endocytosis-related effector or regulator proteins can be used as molecular markers to improve the therapeutic efficacy of gefitinib in lung cancer with wtEGFR. To demonstrate the hypothesis, we attempted to identify differentially expressed genes related to EGFR endocytosis using a genome-wide gene expression assay in gefitinib-sensitive H358 and -insensitive H1703 cells. We found six downregulated genes and two upregulated genes between these two lung cancer cell lines (Supplemental Table S1). Different expression statuses of selected genes were validated by real time-polymerase chain reaction analysis (Data not shown). Rab GTPases (Rab25 and Rab17) exhibited the most distinct gene expression profile among the selected genes. Basically, Rab25 are key regulators in endosomal trafficking and recycling back to the plasma membrane of EGFR [34]. Therefore, we subsequently monitored the endosomal distribution status of EGFR in H358 and H1703 cells after EGF stimulation. As shown in Figure 5A, EGFR was localized in early and recycling endosomes after EGF stimulation in H358 cells expressing Rab25. In contrast, EGFR was only localized in early endosomes of H1703 cells with no Rab25 expression. To further investigate whether Rab25 expression affects EGFR endocytosis, we transfected siRab25 or the scrambled (Scr) control into H358 cells with Rab25 expression. The change of EGFR endocytosis was analyzed by monitoring the internalized proportion of fluorescent-conjugated EGF by flow cytometry. As shown in Figure 5B, internalized EGF increased and then decreased following gefitinib treatment in Scr-transfected cells. However, this inhibition of EGF internalization by gefitinib was less prominent in siRab25-transfected cells than that in Scr-transfected cells.

Next, to evaluate whether the expression status of Rab25 is associated with the response to gefitinib in lung cancer with wtEGFR, we performed a comparative analysis between Rab25 expression and IC_50_ values to gefitinib in eight lung cancer cell lines. As shown in Figure 5C, gefitinib-insensitive cells (H1703, SNU-2292, Calu-1, H441, SNU-1327 and H522) showed relatively low expression levels of Rab25 compared to that in gefitinib-sensitive cells (H358 and Calu-3) (p < 0.05). These results suggest that Rab25 functions significantly in EGFR endocytosis and the response of gefitinib in lung cancer with wtEGFR.

### Inhibiting Rab25 expression reverses the response to gefitinib in vitro and in vivo

**Figure 6:**
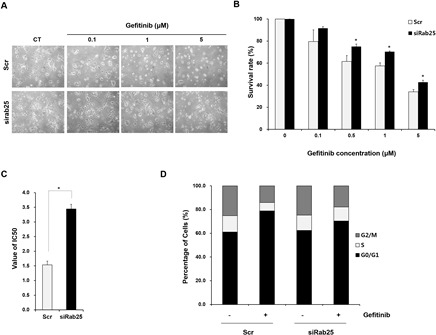
Inhibition of Rab25 expression reverses sensitivity to gefitinib After siRab25 or scrambled (Scr) transfection, H358 cells were treated with the indicated concentrations of gefitinib. (A) Changed gefitinib response based on Rab25 expression status was monitored by light microscopy. Cell images were captured at a magnification of 100×. Cell viability (B) and IC_50_ (C) in siRab25 or Scr transfected cells were determined after gefitinib treatment using MTT assay. Each data point represents mean results of six independent determinations with standard error. * p < 0.05. (D) Redistributed percentages of the cell cycle caused by Rab25 expression status were analyzed after gefitinib treatment using flow cytometry. Data represents mean results acquired from three independent experiments.

To demonstrate whether regulating Rab25 expression affects sensitivity to gefitinib, we tranfected siRab25 or Scr into gefitinib-sensitive H358 cells expressing Rab25. As shown in Figure. 6*a*, morphological changes induced by gefitinib were relatively reduced in siRab25-transfected cells compared with those in Scr-transfected cells.The inhibitory effect of gefitinib on cell viability also decreased significantly in siRab25-transfected cells compared to those in Scr-transfected cells (Figure 6B and C). Moreover, induction of G0/G1 cell cycle arrest caused by gefitinib was smaller in siRab25-transfected cells than that in Scr-transfected cells (Figure 6D).

**Figure 7:**
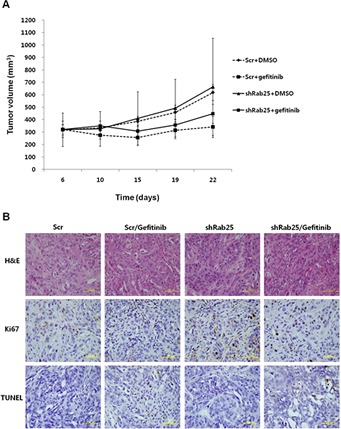
Evaluation of gefitinib response according to Rab25 expression status in a xenograft mouse model (A) Gefitinib-sensitive H358 cells were inoculated subcutaneously after stable shRab25 or shScrambled (Scr) transfection. The mice were treated with DMSO, or gefitinib (5 mg/kg, 4 days/week) intraperitoneally for 2 weeks, and mean tumor volume with standard error was plotted. (B) Hematoxylin and eosin (H&E), Ki67 and TUNEL staining of representative tumor sections from each mouse group. Yellow scale bar is 100 μm.

To further investigate the changes in the gefitinib response by inhibiting Rab25 expression *in vivo*, H358 cells stably transfected with shRab25 or shScrambled control (Scr) were injected subcutaneously into athymic nude nu/nu mice. When the average size of tumors reached 100-200 mm^3^, the mice were divided into four groups: Scr/DMSO, Scr/gefitinib, shRab25/DMSO, and shRab25/gefitinib, and treated intraperitoneally with gefitinib or DMSO. As shown in Figure 7A, although tumor growth in the shScr/gefitinib and shRab25/gefitinib mice groups was delayed after gefitinib treatment, the inhibitory effect of tumor growth caused by gefitinib was higher in the shScr/gefitinib mice groups than in the shRab25/gefitinib mice groups. In addition, the anti-proliferative effect and apoptosis induced by gefitinib was relatively reduced in shRab25/gefitinib mice groups compared to those in shScr/gefitinib mice groups (Figure 7B). These results support the above findings that the status of Rab25 expression is associated with the response to gefitinib in lung cancer with wtEGFR.

## DISCUSSION

In this study, we described a novel association between gefitinib, one of the EGFR-TKIs, and EGFR endocytosis in the treatment of lung cancer with wtEGFR using *in vitro* and *in vivo* models. Gefitinib was initially developed because improper activation of EGFR signaling appears frequently in lung cancer [2, 3]. However, contrary to expectations, the therapeutic efficacy of gefitinib was observed at a very low frequency in all patients with lung cancer, and the improved survival benefit of gefitinib treatment was mostly detected in the specifically selected patients with lung cancer who have EGFR mutations [23]. In other words, patients with wtEGFR, which includes most of the patients with lung cancer, could not expect treatment efficacy from gefitinib. The pharmacological activity of gefitinib is derived from suppressing phosphorylation-mediated TK activity in the EGFR signaling pathway [9]. However, some reports showed disputable results that growth inhibition does not occur in gefitinib-insensitive cells, although EGFR phosphorylation is blocked by gefitinib [35-37]. We also confirmed that EGFR phosphorylation was suppressed after gefitinib treatment in all lung cancer cell lines with wtEGFR regardless of responsiveness to gefitinib. Furthermore, the mutual relationship between the gefitinib response and the oncogenic changes of EGFR such as gene amplification and overexpression remains controversial. Therefore, solely targeting EGFR phosphorylation may not be sufficient to inhibit EGFR-mediated signaling in lung cancer without EGFR mutations, indicating that we do not fully understand EGFR signaling in lung cancer. Based on previous reports and our findings, we attempted to identify other EGFR-related cellular mechanisms in lung cancer with wtEGFR that were associated with cell survival and the gefitinib response independently of its TK activity.

Interestingly, we observed that EGF-induced EGFR endocytosis progressed continuously in gefitinib-insensitive cells regardless of gefitinib treatment, whereas it was suppressed by gefitinib in gefitinib-sensitive cells. We also demonstrated that the difference between these cell lines was correlated with EGFR endocytosis. Although EGFR endocytosis caused by ligand binding has been generally known as a process of down-regulation and degradation for its signal activation, EGFR was occasionally localized in intracellular organelles such as mitochondria and nucleus for cell survival and growth [30, 31]. Furthermore, EGFR signaling is also regulated by the endosomal trafficking before receptor degradation via interaction with various adaptors or signaling molecules [25, 29]. Thus, EGFR endocytosis might be a potent mechanism which contributes to the therapeutic limitation of gefitinib in gefitinib-insensitive lung cancer with wtEGFR. Evidence supporting this hypothesis was detected during endocytosis of Met which is one of the receptor tyrosine kinases (RTKs) with EGFR [38]. That research showed that oncogenic changes of Met endocytosis could be caused by mutations in its kinase domain leading to increased tumorigenicity. Indeed, obstructing Met endocytosis with inhibitors results in tumor regression and reversion of the Met-TKI response. Surprisingly, we also found that suppressing EGFR edndocytosis with inhibitors (dyansore and dynole 34-2) resulted in reduced cell viability and increased apoptotic cell death in gefitinib-insensitive lung cancer cells with wtEGFR, and that inhibitory effect was more enhanced in combination with gefitinib. In particular, dynasore showed a significantly enhanced anti-proliferative effect and apoptotic cell death in mice xenografted with gefitinib-insensitive lung cancer cells with wtEGFR. These results suggest that EGFR endocytosis is a rational target for treating lung cancer with wtEGFR and the efficacy of the combination of its inhibitor and gefitinib is expected.

The molecular mechanism regulating the EGFR endocytic pathway is very complex and tightly controlled by a variety of proteins [25, 33] However, dysfunction of these mediators is currently implicated in human cancers [24, 39]. Therefore, we hypothesized that changed expression of genes related to EGFR endocytosis might be concerned with the different response of gefitinib treatment in lung cancer with wtEGFR. Using a genome-wide expression assay, we identified differentially expressed genes between gefitinib-sensitive and -insensitive lung cancer cells. Based on their biological processes and molecular functions, Rab25 was selected as a potential molecular factor associated with the response to gefitinib treatment. Rab small GTPases including Rab25 play an important role in intracellular vesicle trafficking of RTKs [40]. Rab25, a member of the RAB11 subfamily, specifically controls the return of internalized RTKs to the cell surface [41]. The relationship between the role of Rab25 and tumorigenesis remains controversial. Oncogenic gene amplification and overexpression of Rab25 have been detected in ovarian and breast cancer [42], whereas they function as a tumor suppressor in colon cancer [43]. However, the precise role of Rab25 in lung cancer remains unclear. In the present study, we determined that Rab25 regulated EGFR endocytosis, and its expression status was significantly correlated with the response of gefitinib in lung cancer with wtEGFR. Moreover, we demonstrated that silencing Rab25 reversed the sensitivity to gefitinib in gefitinib-sensitive lung cancer cells expressing normally. The effects of Rab25 knockdown on tumor proliferation and apoptosis were also confirmed in mice xenografted with gefitinib-sensitive lung cancer cells with wtEGFR. Therefore, our findings indicate that Rab25 plays a crucial role in EGFR signaling in lung cancer with wtEGFR.

Although we confirmed meaningful EGFR endocytosis clues that could contribute to treating lung cancer without appropriate therapeutic options, this study had some limitations. First, there is no drug which specifically targets EGFR endocytosis and is approved for clinical use. Actually, the endocytosis inhibitors used in this study inhibit the internalization of other RTKs as well as EGFR, because the RTK endocytosis mechanism exists in the cell membrane and is a common process induced after binding of their ligands [44]. However, EGFR endocytosis is the most reasonable target among the RTKs as oncogenic changes from EGFR signaling mostly occur lung cancer compared to those of other RTK signaling. Second, we did not evaluate Rab25 function in patients with lung cancer and wtEGFR who did not have a response to gefitinib. Therefore, further studies are needed to investigate the relevance of Rab25 in clinical samples.

In conclusion, we provided novel insights that EGFR endocytosis is important for treating lung cancer with wtEGFR in *in vitro* and *in vivo* models, suggesting that it is a promising target to improve therapeutic efficacy when its inhibitor and EGFR-TKI are administered together. In addition, we identified that EGFR endocytosis-related Rab25 expression is crucially considered when patients with lung cancer and wtEGFR are treated with gefitinib.

## MATERIALS AND METHODS

### Reagents

The following reagents and antibodies were used: gefitinib (AstraZeneca, Macclesfield, UK); EGF and fluorescent conjugated EGF (Alexa Fluor 488 and Texas Red) (Molecular Probes (Eugene, OR); dynasore (Sigma) and dynole 34-2 (Ascent Scientific, Bristol, UK). Phospho-AKT (Ser473), AKT, phospho-ERK (Thr202/Tyr204), ERK, poly (ADP-ribose) polymerase (PARP), Mcl-1, XIAP, survivin, livin, Rab17 and Rab25 (Cell Signaling Technology, Beverly, MA); phospho-EGFR (Tyr1173), EGFR, normal mouse IgG, secondary donkey anti-goat IgG-HRP and goat anti-rabbit IgG-HRP (Santa Cruz Biotechnology, Santa Cruz, CA); β-actin (Sigma); goat anti-mouse IgG (H + L)-HRP conjugate (Bio-Rad); Alexa Fluor® 488 goat anti-mouse IgG (H+L) (Molecular Probes, Eugene, OR); EEA1, CD63 and Rab11 (BD Biosciences, Erembodegem, Belgium).

### Cell lines and cell culture

The lung adenocarcinoma cell lines (Calu-3, H441, and H522), squamous cell carcinoma cell lines (H1703 and Calu-1), and the bronchial alveolar carcinoma cell lines (H358) were obtained from the American Type Culture Collection (Rockville, MD). Additional lung adenocarcinoma cell lines (SNU-1327 and SNU-2292) were obtained from the Korean Cell Line Bank (Seoul, South Korea). H358, H1703, Calu-1, H441, H522, SNU-1327, and SNU-2292 cells were cultured in RPMI-1640 medium supplemented with 10% fetal bovine serum (FBS), and 1% penicillin/streptomycin. Calu-3 cells were cultured in Eagle's Minimum Essential Medium supplemented with 10% FBS, and 1% penicillin/streptomycin. All cultured cells were incubated in a humidified 37°C incubator with 5% CO_2_.

### MTT assay

After drug treatment, cells were mixed with 20 μl of 3-(4,5-dimethylthiazol-2-yl)-2,5-diphenyltetrazolium bromide (MTT) (Sigma) solution (5 mg/ml in 1x PBS) in each well and further incubated for 4 h at 37°C. The medium was removed and 200 μL of dimethyl sulfoxide (DMSO) (Sigma) was added in each well. Absorbance was detected at 540 nm with an iMark microplate reader (c). Results are presented as a relative percent surviving normalized to untreated control cells. The experiment was repeated independently three times. IC_50_ values were determined using the microplate reader software Softmax Pro (Molecular Devices Cooperation Corp., Sunnyvale, CA).

### Western blotting analysis

Briefly, cells were harvested and washed with 1× PBS and lysed with lysis buffer (10 mM Tris-HCl pH 8.0, 1 mM EDTA, 150 mM NaCl, and 0.2% Triton X-100) containing a protease inhibitor and phosphatase inhibitor cocktail (GenDEPOT, Seoul, Korea). Protein concentration was determined using the Bradford method with bovine serum albumin (BSA) as the standard (Sigma). The protein sample (30 μg) was run on 8-12% sodium dodecyl sulfate-plyacryamide gel electrophoresis and transferred to PVDF membranes (Amersham). The membranes were blocked and further incubated overnight at 4°C with appropriate primary antibodies. The membranes were washed and incubated for 1 h at 4°C with appropriate secondary antibody and visualized on X-ray film using the ECL Western Blotting Detection Reagents (Amersham). Band density was analyzed with Quantity-one™ image analysis software (Bio-Rad).

### Propidium iodide (PI) staining

The cells were harvested and washed twice with cold 1× PBS, then fixed in cold 70% ethanol for 2 h at 4°C. After the ethanol solution was removed, the cells were stained with PI staining solution (40 μg PI/100 μg RNase A/1 ml PBS) for 1 h at 37°C. The status of PI- stained DNA content was analyzed using FACS Calibur and Cellquest software (Becton Dickinson). The percentage of cells in the sub-G1, G1, S, and G2/M phases was analyzed with WinMDI 2.9 software (The Scripps Institute, La Jolla, CA).

### EGFR phosphorylation antibody array

The phosphorylation status of 10 different EGFR sites was simultaneously detected by EGFR phosphorylation antibody array (RayBiotech, Norcross, GA), according to the manufacturer's instructions. The membranes were exposed to photographic X-ray films. Signal intensity was quantified by Quantity-one™ image analysis software (Bio-Rad). The relative expression levels of the phosphorylated EGFR sites were normalized to total EGFR signal in each membrane.

### Immunofluorescence staining

Cells were cultured on 18-mm cover glasses and washed with ice-cold 1× PBS. After drug treatment, the cover glass was fixed with 4% PFA/1× PBS for 15 min at 37°C, and washed three times with 1× PBS-T, and permeabilized with 0.5% Triton X-100/1× PBS for 30 min at room temperature. All of the following steps were performed in the dark. After blocking with 5% BSA/1× PBS-T, the cover glass was incubated with the appropriated antibodies in 5% BSA/1× PBS-T overnight at 4°C. The next day, the cover glass was washed three times with 1× PBS-T and incubated with fluorescent conjugated secondary antibodies in 5% BSA/1× PBS-T for 1 h at room temperature and couterstained with DAPI (Sigma) for 10 min at room temperature. After washing, the cover glass was dried and mounted using fluorescent mounting medium (Dako, Carpenteria, CA). Fluorescence images of cells were captured under an Olympus BX51 microscope (Olympus, Tokyo, Japan) equipped with DP70 digital camera and DP Manager software.

### Fluorescent-conjugated EGF uptake

EGFR internalization was analyzed by modified flow cytometry with fluorescent-conjugated EGF. Briefly, the cells were washed with 1× PBS after EGF stimulation and further washed with an acid solution (0.5% acetic acid, 0.5 M NaCl, pH 3.0) for 5 min at 4°C. The cells were then trypsinized and resuspended in FACS buffer. The status of fluorescent- conjugated EGF uptake was analyzed using FACS Calibur and Cellquest software (Becton Dickinson). The percentage of cells with internalized EGF was analyzed using WinMDI 2.9 software (The Scripps Institute,. La Jolla, CA).

### Apoptotic cell death assay

Apoptotic cell death was measured with an FITC Annexin V Apoptosis Detection Kit I (BD Biosciences, San Diego, CA), according to manufacturer's protocol. Briefly, the cells were harvested after drug treatment and washed with cold 1× PBS twice, resuspended in 1× binding buffer, and counted at a concentration of 1 × 10^6^ cells/mL. Aliquots (100 μL) of resuspended cells were transferred to a new 1.5 mL tube and incubated with FITC Annexin V (5 μL) and PI (5 μL) for 15 min at room temperature in the dark. Finally, 500 μL of 1× binding buffer was added to each tube and the cells were then analyzed by FACS Caliber (Becton Dickinson, Hertfordshire, UK). The percentage of FITC Annexin V-positive cells was analyzed using WinMDI 2.9 software (The Scripps Institute, La Jolla, CA).

### Whole genome expression assay

A gene expression profile of H358 and H1703 lung cancer cells was obtained using the genome-wide HumanHT-12 v4 Expression Bead Chip arrays (Illumina, San Diego, CA). Briefly, total RNA was isolated using TRIZOL reagent and quantified using an ND-1000 spectrophotometer (Nano-Drop, Wilmington, DE). After RNA amplification, labeling, hybridization, scanning and statistical analysis of samples were performed by Macrogen Inc. (Seoul, Korea). Array data export processing and analysis was performed using Illumina Genome Studio v2009.2. The quality of the hybridization and overall chip performance were monitored by visually inspecting both internal quality control checks and the raw scanned data and normalized by quantiles.

### siRNA and shRNA transfection

Briefly, H1703 cells (2×10^5^ cells/well) were seeded into six-well plates and grown to 80% confluence on the day of transfection. The scrambled siRNA (Scr, 5'-GGC CAG AAC UAG UAC AUC CCG AAC U-3') and siRab25 (5'-GGA GCU CUA UGA CCA UGC U-3') were synthesized at Bioneer (Daejeon, Korea). Transfection of cells with siRNAs was performed using LipofectAMINE 2000 (Invitrogen, Carlsbad, CA, USA) according to the manufacturer's instructions.

Stable knockdown of Rab25 expression was established by shRNA transfection. H358 cells (2 × 10^5^ cells/well) were seeded and incubated for 24 h prior to viral infection. After replacing the culture medium with a Polybrene/media mixture, the cells were incubated with shScrambled control (Scr) and Rab25 shRNA lentiviral particles (Santa Cruz Biotechnology, Santa Cruz, CA) overnight. The next day, the culture medium was replaced with complete growth medium, and stable clones expressing shRNA were selected via further incubation with puromycin (1 μg/mL).

### Animal studies

Male athymic nude (nu/nu) mice (20g, 6 weeks old) were purchased from Nara Biotech (Seoul, Korea). Lung cancer SNU-1327 (5 × 10^6^ cells) and H358 (1 × 10^7^ cells) were injected subcutaneously into the flanking regions of mice together with Matrigel (1:1) (BD Biosciences, Erembodegem, Belgium). When the tumor reached a size of 100-200 mm^3^, the mice were treated intraperitoneally with appropriate drugs (details in the Figureure legends) and randomly divided into four groups (five mice per group). Tumor diameters were measured with calipers twice per weekly for ≥ 5 weeks. Tumor volumes were calculated with the following formula: volume (mm^3^) = width^2^ × length/2. The mice were sacrificed, and tumor tissues were collected for histological analysis. Hematoxylin and eosin stain or immunohistochemistry staining was performed on the tumor sections. Immunostaining for Ki67 was carried out using the Polink-2 HRP Plus Broad DAB Detection System (GBI Labs, Mukilteo, WA) with a monoclonal mouse antibody to human Ki67 (Dako, Carpenteria, CA). Apoptotic cells were determined by using the terminal deoxynucleotidyl transferase dUTP nick end labeling assay (Millipore, Temecula, CA) according to the manufacturer's instructions. The stained images were captured under an Olympus BX51 microscope (Olympus, Tokyo, Japan) equipped with DP70 digital camera and the DP Manager software. Staining results were evaluated by pathologists according to the immunodetection of stain intensity. All mice procedures were performed according to the Institutional Animal Care and Use and IRB committees (KUIACUC-2013-80) at the Korea University (Seoul, Korea).

### Statistical analysis

Results are expressed as mean ± standard error. Multiple comparisons were performed using one-way ANOVA with SPSS ver. 12.0 (SPSS Inc. Chicago, IL). Differences were considered significant at *p* < 0.05.

## SUPPLEMENTARY FIGURES AND TABLE




