# Supplementary material for: EGFR endocytosis is a novel therapeutic target in lung cancer with wild-type EGFR
Source: Oncotarget. 2014 Jan 16;5(5):1265–78. doi: 10.18632/oncotarget.1711 (PMC4012721; doi:10.18632/oncotarget.1711)
Supplement: Supplementary file 1 [file oncotarget-05-1265-s001.pdf]

EGFR endocytosis is a novel therapeutic target in lung cancer with wild-type EGFR – Jo et al

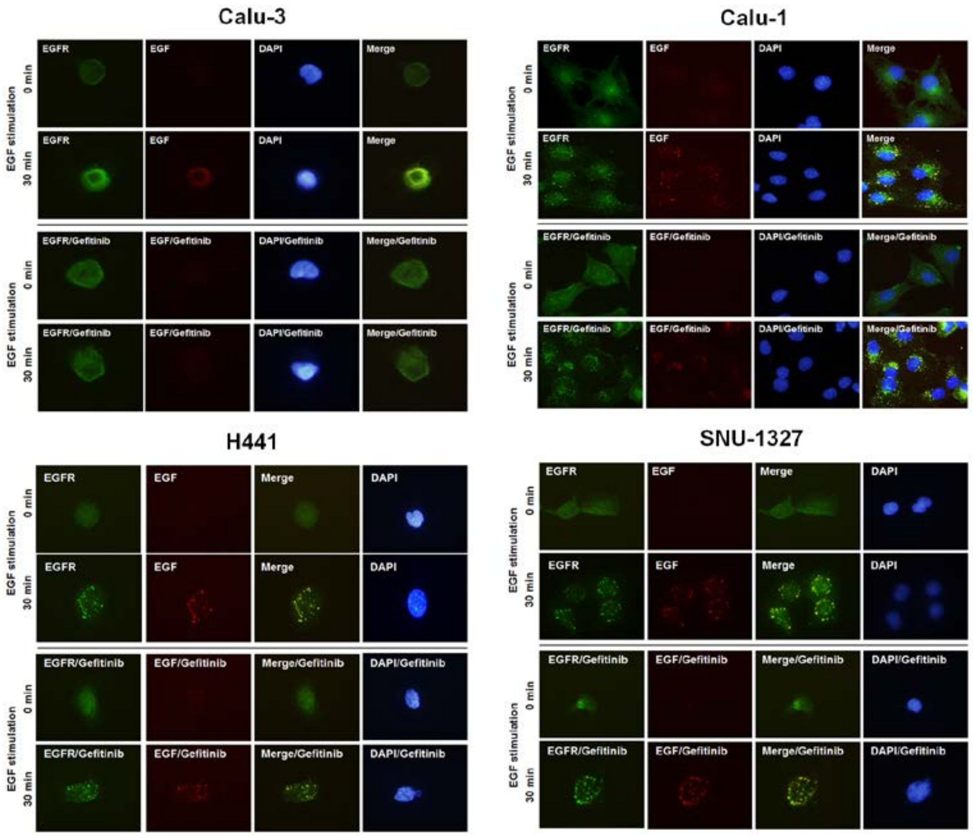

Figure S1: EGFR cellular distribution after gefitinib treatment in other lung cancer cell lines with wtEGFR. The difference in EGF-induced EGFR cellular distribution after gefitinib treatment was profiled in gefitinib-sensitive cell line (Calu-3) and gefitinib-insensitive cell lines (Calu-1, H441 and SNU-1327) by immunoflurescence staining.

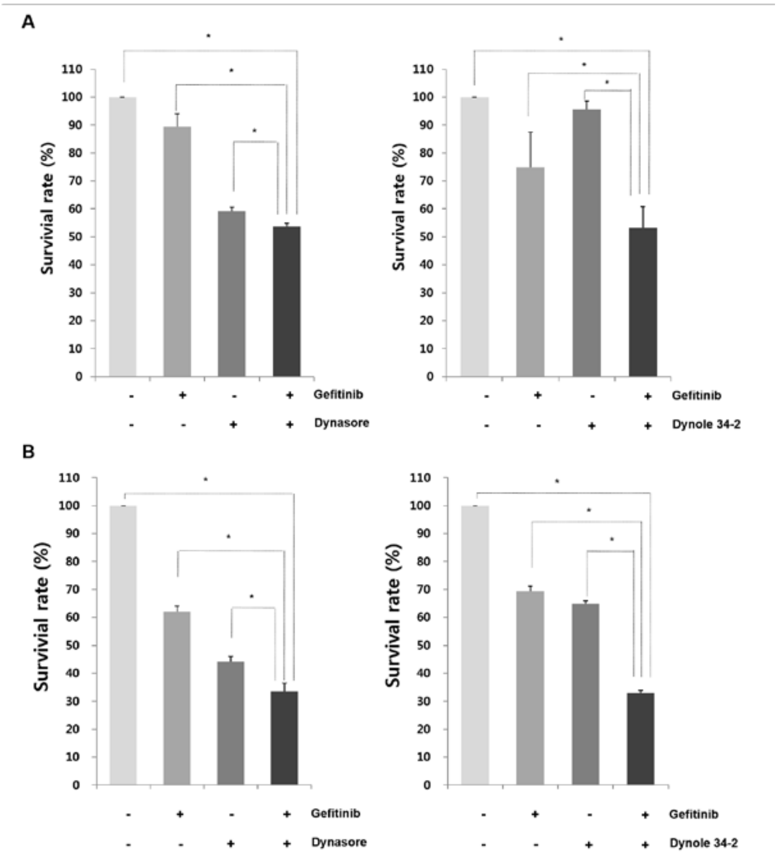

Figure S2: Evaluation of the anti-proliferative effects of EGFR endocytosis inhibitors in other gefitinib-insensitive lung cancer cell lines with wtEGFR. Effects of EGFR endocytosis inhibitors alone or combination with gefitinib on cell viability were analyzed by the MTT assay in Calu-1 (A) and SNU-1327 (B) cells. The cells were treated with dynasore (100 μM) or dynole 34-2 (10 μM) or in combination with gefitinib (10 μM) for 48 h. Each bar represents mean values acquired from three experiments with standard error. * $p < 0.05$.
