# Supplementary material for: EGFR endocytosis is a novel therapeutic target in lung cancer with wild-type EGFR
Source: Oncotarget. 2014 Jan 16;5(5):1265–78. doi: 10.18632/oncotarget.1711 (PMC4012721; doi:10.18632/oncotarget.1711)
Supplement: Supplementary file 2 [file oncotarget-05-1265-s002.pdf]

Supplemental Table

Supplemental Table S1. List of genes differentially expressed between gefitinib-sensitive and -insensitive lung cancer cells with wtEGFR

Expression	GeneSymbol	Fold change	H1703/H358.Lstat	H1703/H358.Ladjp
Down	Rab17	-23.60	-23.95	3.12E-125
Down	Rab25	-20.82	-24.04	3.37E-126
Down	RIN2	-9.08	-16.36	9.50E-59
Down	SPRY1	-5.70	-12.94	0.00
Down	DNM3	-5.58	-12.83	2.35E-36
Down	MUC1	-2.35	-6.46	0.00
Up	SGK1	48.13	33.76	0.00
Up	ECH1	29.97	28.35	0.00
